# The *C*^1^*C*^2^: A framework for simultaneous model selection and assessment

**DOI:** 10.1186/1471-2105-9-360

**Published:** 2008-09-02

**Authors:** Martin Eklund, Ola Spjuth, Jarl ES Wikberg

**Affiliations:** 1 Department of Pharmaceutical Pharmacology, Uppsala University, Box 591, BMC, SE-751 24 Uppsala, Sweden

## Abstract

**Background:**

There has been recent concern regarding the inability of predictive modeling approaches to generalize to new data. Some of the problems can be attributed to improper methods for model selection and assessment. Here, we have addressed this issue by introducing a novel and general framework, the *C*^1^*C*^2^, for simultaneous model selection and assessment. The framework relies on a partitioning of the data in order to separate model choice from model assessment in terms of used data. Since the number of conceivable models in general is vast, it was also of interest to investigate the employment of two automatic search methods, a genetic algorithm and a brute-force method, for model choice. As a demonstration, the *C*^1^*C*^2 ^was applied to simulated and real-world datasets. A penalized linear model was assumed to reasonably approximate the true relation between the dependent and independent variables, thus reducing the model choice problem to a matter of variable selection and choice of penalizing parameter. We also studied the impact of assuming prior knowledge about the number of relevant variables on model choice and generalization error estimates. The results obtained with the *C*^1^*C*^2 ^were compared to those obtained by employing repeated *K*-fold cross-validation for choosing and assessing a model.

**Results:**

The *C*^1^*C*^2 ^framework performed well at finding the true model in terms of choosing the correct variable subset and producing reasonable choices for the penalizing parameter, even in situations when the independent variables were highly correlated and when the number of observations was less than the number of variables. The *C*^1^*C*^2 ^framework was also found to give accurate estimates of the generalization error. Prior information about the number of important independent variables improved the variable subset choice but reduced the accuracy of generalization error estimates. Using the genetic algorithm worsened the model choice but not the generalization error estimates, compared to using the brute-force method. The results obtained with repeated *K*-fold cross-validation were similar to those produced by the *C*^1^*C*^2 ^in terms of model choice, however a lower accuracy of the generalization error estimates was observed.

**Conclusion:**

The *C*^1^*C*^2 ^framework was demonstrated to work well for finding the true model within a penalized linear model class and accurately assess its generalization error, even for datasets with many highly correlated independent variables, a low observation-to-variable ratio, and model assumption deviations. A complete separation of the model choice and the model assessment in terms of data used for each task improves the estimates of the generalization error.

## Background

A common task in computational biology/bioinformatics and computational chemistry/chemometrics (hereafter abbreviated BBCC) is to model a dependent variable from a set of independent variables; this gives insight into the workings of the process being modeled and enables prediction of future observations. Typical examples include analyzing potential drug activity through proteochemometrics and quantitative structure-activity relationship (QSAR) modeling [1-3], discovering gene regulatory binding-site modules [4], and predicting clinical outcomes of cancer from gene expression data [5]. However, recent articles have indicated that predictive modeling approaches have not fully fulfilled expectations for solving real problems. This issue has for instance been discussed in the fields of QSAR [6] and microarray gene expression data modeling [7,8]. While some of the problems may be attributed to incorrect use and interpretation of the models, others can be ascribed to improper model selection and assessment. Our aim is here to address the latter issue by introducing the *C*^1^*C*^2^, a general framework for model choice and assessment.

Let ***D ***= {***X***_*n*_, ***y***_*n*_} be a dataset, where ***X***_*n *_= (***x*'**_1_, ..., ***x*'**_*n*_)' is an *n *× *p*_*X *_matrix whose *i*th row, ***x***_*i*_, is the value of a *p*_*X*_-vector of independent variables associated with *y*_*i*_, the *i*th row of the *n *× 1 matrix, ***y ***= (*y*_1_, ..., *y*_*n*_)'. A statistical model, $M_{l}$ can be used to characterize the relation between ***X***_*n *_and ***y***_*n*_. In general, given the dataset ***D***, $M_{l}$ must be chosen from a set of models, $M=\left\{M_{1},...,M_{m},...,M_{M}\right\}$ according to some criterion (typically the minimization of a loss function). The most common way to select $M_{l}$ from M in BBCC is to use *K*-fold cross-validation; that is, the dataset ***D ***is split into *K *mutually exclusive subsets, ***D***_1_,..., ***D***_*k*_,..., ***D***_*k*_, of approximately equal size and $M_{l}^{0}$ (***D***) is picked to minimize the function:

(1) $$C^{0}(M_{m})=\frac{1}{n}\sum_{k=1}^{K}\sum_{i=1}^{n_{D_{k}}}L(y_{i},{\hat{y}}_{i}(x,M_{m},D_{-k})),$$

where *L *is a loss function and ***D***_-*k *_denotes that the *k*th subset was excluded from ***D ***during the model fitting; $n_{D_{i}}$ is the number of observations in subset *D*_*i*_; and *m *= 1,..., *M*, where *M *is the number of models in M. This model selection method may seem straightforward and intuitive, however it neglects the fact that all the data at hand is used to make the model choice. Thus, we no longer have an independent testset to assess the chosen model by. The result is that, typically, *C*^0^($M_{l}$) underestimates the generalization error (see for instance [9]), defined as the expected prediction error over an independent test sample. This problem has been highlighted in relatively recent works [9,10], but was noted initially in 1974 [11]. To obtain a more accurate generalization error estimate, the model selection process must be separated from the model assessment in terms of the data that is used. Ideally, if data were abundant and easily produced, we would set aside a large test dataset and use it to assess – but not to choose! – the model $M_{l}$, and subsequent model refinements could be assessed with new, unseen data. In practice, this is however often impossible since BBCC data is typically scarce, and expensive to produce. The luxury of large independent testsets can thus rarely be afforded. To tackle this problem, Freyhult et al. [9] suggested using a *K*-fold cross-validatory assessment of an *H*-fold cross-validatory model choice, $M_{l}^{†}$, as a way of simultaneously choosing $M_{l}$ and assessing its performance; thereby separating the model selection from its assessment. The model is assessed by the function

(2) $$C^{†}=\frac{1}{n}\sum_{k=1}^{K}\sum_{i=1}^{n_{D_{k}}}L(y_{i},{\hat{y}}_{i}(x_{i},M_{l}^{†}(D_{-k}),D_{-k})),$$

where $M_{l}^{†}$ (***D***_-*k*_) is the cross-validatory choice of $M_{l}$ based on ***D***_-*k*_; that is, the model $M_{l}$ that minimizes the function

(3) $$C_{D_{-k}}^{†}(M_{m})=\frac{1}{n_{D_{-k}}}\sum_{h=1}^{H}\sum_{j=1}^{n_{D_{h}}}L(y_{j},{\hat{y}}_{j}(x_{j},M_{m},D_{-hk})),$$

where ***D***_-*hk *_denotes the dataset ***D ***with the *k*th and *h*th subsets omitted. In the present work we build on and generalize this idea into the *C*^1^*C*^2 ^framework. In general, the number of models in M is huge, thus it is unfeasible to go through even a small subset of them manually. Hence, for a framework such as the *C*^1^*C*^2 ^to be useful in practice, automated methods for searching the model space M are necessary; in this sense the *C*^1^*C*^2 ^is similar to the automatic modelling approaches taken in for instance [12-14]. Here, the specific use of the *C*^1^*C*^2 ^is demonstrated by applying it together with two search methods to simulated and real-world datasets. The results are compared to those obtained by employing the function (1) for model selection and assessment. In the interest of clarity, we have restricted our attention to the study of model choice and assessment within a linear model class, $M^{ridge}$ (defined below) for a quadratic loss function. We discuss the results of the demonstrations, the pros and cons of the generality of the *C*^1^*C*^2^, and set out some directions for further research.

## Results

### Algorithm

Let *C*^1^, *C*^2 ^∈ ***C ***= {*C*_1_,..., *C*_*J*_}, where ***C ***is a set of model assessment criteria and *C*^1^, *C*^2 ^represent two specific criteria (i.e. *C*^1 ^= *C*_*i*_, *C*^2 ^= *C*_*j*_, *i*, *j *= 1,..., *J*). Further, let *S *∈ ***S***, where ***S ***is a set of search methods; let *L *∈ ***L***, where ***L ***is a set of loss functions; let ***G ***denote a sequence of data processing steps (e.g. mean-centering, transformations, whitening, etc) and let ***G****' *contain the results of ***G ***applied to ***D***_-***k ***_(the roles of ***G ***and ***G****' *are exemplified in the discussion following the pseudococe below); let *R *be a positive integer and *K *an integer between 1 and *n*, where *n *is the number of observations. The *C*^1^*C*^2 ^procedure is outlined with the following pseudocode:

Initiate M, ***G***, *L*, *C*^1^, *C*^2^, *R*, and *K*.

for (*r *in 1,..., *R*) {

   a. Partition data, ***D ***= {***D***_*k*_}_*k *= 1,..., *k*_.

   for (k in 1,..., *K*) {

      b. Apply ***G ***to ***D***_-*k*_. Save results in ***G***'.

      c. Search M using the data ***D***_-*k *_and *C*^2 ^as objective function. Assume $M_{l}$ is found to maximize (or minimize) *C*^2^. Save $M_{l}$.

      d. Apply ***G ***to ***D***_*k *_using ***G***'.

      e. Assess $M_{l}$ using *C*^1 ^and ***D***_*k*_. Save assessment result.

   }

}

The data partitioning in (a) separates data for the model choice from data for the model assessment. Note that the partitioning is dependent on the choice of *C*^1 ^and does not necessarily need to be done in a cross-validation fashion. For instance, the choice *C*^1 ^= ".632 estimator" [15,16], partitions the data by independently sampling *n *rows from ***D ***with replacements and lets the observations not included among the sampled observations constitute the test set. The output from the *C*^1^*C*^2 ^is also dependent on the choice of *C*^1^; for example, the choice *C*^1 ^= *C*^2 ^= Bayesian Information Criterion (BIC, see [17] and Methods) would not give a direct estimation of the generalization error, but rather an assessment of model overfitting. To clarify the roles of ***G ***and ***G'***, we give the following example: Let ***G ***only contain a processing step that scales to unit variance. In (b) ***G ***is applied to ***D***_-*i *_and the standard deviation of each column of ***D***_-*k *_is saved in ***G*'**. In (d), ***G*' **is applied to ***D***_*k*_, that is, the columns in ***D***_*k *_are scaled using the standard deviations calculated in (b). This treatment of ***G ***ensures that ***D***_*k *_indeed constitutes an independent testset. The 'for loop' over *r *is introduced to enable calculation of confidence intervals for estimates and, by averaging the estimates over *R *repetitions, it permits reduction of the variance in parameter and error (or overfitting) estimates by a factor of 1/*R*.

**Figure 1:**
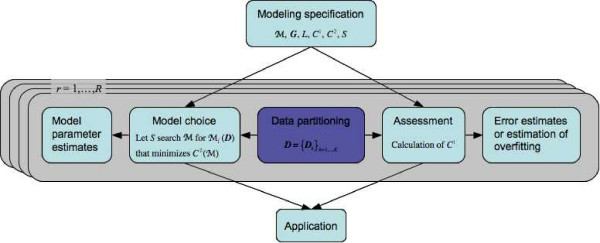
**The *C*^1^*C*^2 ^framework**. The data partitioning in step (a) in the pseudocode separates the model choice from its assessment, which is highlighted in purple in the figure. The left side of the figure relates to steps (b) to (d) in the pseudocode, and the right side to step (e); i.e. the left side relates to choosing the model and saving the parameter estimates, and the right side to assessing the model and saving the assessment results.

Figure 1 gives a graphical gives a graphical view of the *C*^1^*C*^2 ^framework. We emphasize that the generality of the framework allows *C*^1^, *C*^2^, and *S *to be chosen to fit the problem at hand. Adequate choices of *C*^1^, *C*^2^, and *S *make the model selection and assessment more accurate and faster, which we will discuss below.

### Datasets used in the testing

Both the simulated and the real data used for evaluating a new method or algorithm should reflect typical dataset properties found in real-world application domains. Examples of such properties in BBCC are multicollinearity, a large number of independent variables relative to the number of observations, and binary and categorical independent variables.

#### Simulated data

We simulated datasets as follows:

Let Δ = (*δ*_*i*_)_*i *= 1,..., *p*_, where $\delta_{i}=\{\begin{array}{l} 1\text{ iff }x_{i}\text{ is in the model}\\ 0\text{ else} \end{array}$, represents a subset of ***X***_*n*_, and let *β*_*p*_(Δ) = (*β*_*i*_(*δ*_*i*_))_*i *= 1,..., *p*_, where $\beta_{i}(\delta_{i})=\{\begin{array}{l} \beta_{i}\neq 0\text{ iff }\delta_{i}=1\\ 0\text{ else} \end{array}$, is a vector of regression coefficients. The data matrix, ***X***_*n *_was sampled from a twenty-dimensional multivariate normal distribution. Thus, ***x***_*i *_~ *N*_20_(**0**_20_, Σ_20_), *i *= 1,..., *n*, where **0**_20 _is a twenty-dimensional vector of zeroes and Σ_20 _is either the ***I***_20 _identity matrix or a covariance matrix, ***S***_20_, estimated from a real in-house QSAR dataset that originated from HIV protease inhibitors. The HIV QSAR dataset contains highly correlated independent variables resulting in an ***S***_20 _with many large absolute values in the off-diagonal elements. Three *δ*_*i *_in Δ were chosen to be nonzero and equal to one; the positions were chosen at random to be 11, 14, and 18 (but remained fixed throughout the experiment for evaluation purposes). The corresponding regression coefficients, *β*_11_(*δ*_11_), *β*_14_(*δ*_14_), and *β*_18_(*δ*_18_), were, for simplicity, all set equal to 1. The variables 11, 14, and 18 were slightly correlated with an estimated covariance matrix $\Sigma_{\left(11,14,18\right)}=\left(\begin{array}{ccc} \text{4}\text{.07} & \text{15}\text{.28} & \text{-0}\text{.01}\\ \text{15}\text{.28} & \text{3423}\text{.20} & \text{-0}\text{.33}\\ \text{-0}\text{.01} & \text{-0}\text{.33} & \text{0}\text{.01} \end{array}\right).$.

Datasets were generated assuming that ***y***_*n *_followed a linear model according to: ***y***_*n *_= ***X***_*n *_*β*_*p*_(Δ) + *ε*_*n*_, where *ε*_*i*_~*N*(0,1.5). Four datasets were simulated in order to evaluate the *C*^1^*C*^2^s performance in settings where *n *<*p*_*x*_, *n *> *p*_*x*_, and where the observations were sampled from an orthogonal multivariate normal distribution or not, according to the following schema:

1. *n *= 15, Σ_20 _= ***I***_20_

2. *n *= 200, Σ_20 _= ***I***_20_

3. *n *= 15, Σ_20 _= ***S***_20_

4. *n *= 200, Σ_20 _= ***S***_20_

The simulated datasets are available in CSV format from Additional files 1, 2, 3, 4.

#### The Selwood dataset

This is a real dataset, made available from a website [18] and originally published in 1990 [19]. It is a widely studied dataset (see [20,21] and references therein). It contains one dependent variable, 53 independent variables, and 31 observations. The 53 independent variables correspond to numerical descriptions of molecules (antifilarial antimycin analogues) designed to capture their physicochemical properties. The dependent variable is the *in vitro *antifilarial activity of the molecules. This dataset exhibits extremely strong correlations between the independent variables and contains real valued, binary and categorical independent variables. It is known from previous studies that this dataset contains nonlinearities, but that decent models can be found using linear methods.

### Testing

To demonstrate the use of the *C*^1^*C*^2^, it was applied to the simulated and real-world datasets described above (hereafter referred to as "the datasets"). Below we describe the choices for *R*, *K*, M, ***G***, *L*, *C*^1^, *C*^2^, and *S *and the motivation for each selection.

#### Choice of *R *and *K*

The larger the choice of *R*, the higher the accuracy in the estimate of the generalization error; the choice of *R *is thus constrained by time and is dependent on the size of the dataset and the computational complexity of the choices of M, ***G***, *L*, *C*^1^, *C*^2^, and *S*. The choice of *K *is a trade-off between bias and variance; the larger the *K*, the more variance and the less bias in the estimates of the generalization error [22]. *R *was here set to 12 and *K *to 5.

#### Choice of M

We make the assumption that a normal linear model forms a reasonable approximation of the data. This model is given by: *y*_*n *_= ***X***_*n *_*β*_*p *_+ *ε*_*n*_, where the subscripts denote the number of rows in a matrix, *β*_*p *_are regression coefficients, and *ε*_*n*_~*N*(0,*σ*^2 ^***I***). Further, because *n *<*p *and the columns in ***X***_*n *_are highly correlated in some of the datasets, we decide to use the ridge estimator, ${\hat{\beta }}_{p}^{ridge}$ (see Methods), of the regression coefficients, *β*_*p*_. Let $M^{ridge}$ be the linear class of models given by: ***y***_*n *_= ***X***_*n *_*β*_*p *_+ *ε*_*n*_, where *β*_*p *_is estimated by ${\hat{\beta }}_{p}^{ridge}$. We thus choose $M=M^{ridge}$. The problem of model choice within $M^{ridge}$ reduces to the problem of variable selection, i.e. choosing which subset of the *p *columns in ***X***_*n *_to include in the model, and the problem of choosing the ridge penalizing parameter *λ *(see Methods). Hence, letting Δ = (*δ*_*i*_)_*i *= 1,..., *p *_(see simulated data above) represent a subset of ***X***_*n*_, we want to choose Δ and *λ *using the *C*^1^*C*^2 ^framework. A choice of Δ and *λ *for given values of *r *and *k *will be termed "an estimate" of Δ and *λ*, respectively, and be denoted $\hat{\Delta }$ and $\hat{\lambda }$. Averages of estimates over the *K *folds and the *R *repeats in the *C*^1^*C*^2 ^are denoted $\bar{\hat{\Delta }}$ and $\bar{\hat{\lambda }}$, respectively.

#### Choice of *G*

As the columns in the Selwood dataset are measured in different units using different scales, we choose to make ***G ***contain mean centering and scaling to unit variance processing steps.

#### Choice of *L*

We use the standard quadratic loss function given by:

$$L(y_{i},{\hat{y}}_{i}(x_{i},M_{m}))={\left(y_{i}-{\hat{y}}_{i}(x_{i},M_{m})\right)}^{2}.$$

#### Choice of *C*^1 ^and *C*^2^

Others [12,13] have suggested choosing *C*^1 ^= *C*^2 ^= cross-validation. Here, we choose *C*^1 ^= cross-validation and *C*^2 ^= BIC. Hence, in this demonstration we assess a model choice $M_{l}^{ridge}\in M^{ridge}$ according to:

(4) $$C^{1}=\frac{1}{n}\sum_{k=1}^{K}\sum_{i=1}^{n_{D_{k}}}L(y_{i},{\hat{y}}_{i}(x_{i},M_{l}^{ridge,BIC}(D_{-k}),D_{-k})),$$

where $M_{l}^{ridge,BIC}$ (***D***_-*k*_) is the $M_{l}^{ridge}$ chosen according to BIC based on ***D***_-*k*_; that is, the value of $M_{l}^{ridge}$ that optimizes the function:

(5) $$C_{D_{-k}}^{2}(M_{m}^{ridge})=\log\text{P}(D_{-k}|{\hat{\beta }}^{ridge},M_{m}^{ridge})-\frac{df}{2}\log n_{D_{-k}},$$

*m *= 1,..., *M*, where *M *is the number of models in $M^{ridge}$. *df *in (5) is the number of free parameters in the model $M_{m}^{ridge}$ (note that this is not equal to the number of parameters in the model $M_{m}^{ridge}$, see for instance [16]). The choice of *C*^1 ^is motivated by that we wish to get a direct estimate, ${\hat{\varepsilon }}_{gen}$, of the generalization error, *ε*_*gen*_, of our model choice. Provided that the assumptions behind BIC are fulfilled, the choice *C*^2 ^= BIC has several advantages over *C*^2 ^= cross-validation, including: a reduction of bias in parameter estimates [22], a reduction of variance by the Rao-Blackwell type relation derived in [23], and a drastic reduction of the computational cost of the procedure.

#### Choice of *S*

A genetic algorithm (GA) was chosen as a search method because it is very easy to adapt to different situations and in general effective for nondeterministic polynomial-time hard combinatorial problems, such as the problem of estimating Δ [24]. A trial solution in the GA is here a varying length chromosome that contains a real-valued number representing *λ *and a vector of integers representing the indices of the *δ*_*i *_in Δ that are nonzero. The fitness function is our choice of *C*^2^. For the simulated datasets, we also chose to run the *C*^1^*C*^2 ^with a brute force search method: for each *λ *∈ {0,0.01,0.02,...,10} an exhaustive search in the Δ space (i.e. an all-subset regression) was performed. This enabled comparisons between the GA method and a search method guaranteed to find the optimal model (given a specific objective function and the resolution and limits of *λ*).

### Some remarks regarding the demonstration

To enable comparisons with the estimates of Δ, *λ*, and *ε*_*gen *_obtained with repeated *K*-fold cross-validation, the demonstration described above was repeated with the function (1) used as a criterion for model choice and for assessing the model. Note that, since the *C*^1^*C*^2 ^includes the 'for loop' over *r*, (1) was repeated *R *= 12 times, each time with a new, independent data partitioning. This was done to facilitate an impartial comparison between the two methods.

The demonstration of the *C*^1^*C*^2 ^framework can be compared with the work of for instance Nicolotti and Carotti [20], where a genetic algorithm was employed to estimate Δ. In contrast to that approach, the *C*^1^*C*^2 ^framework completely separates model choice and assessment whereby more accurate generalization error estimates in general are achieved. Further, the use of specific ad hoc objective functions is avoided by choosing *C*^2 ^to be a formally derived model selection criterion, and simultaneous estimation of model parameters other than Δ (for example, the ridge parameter *λ *in the demonstration) can be afforded. Typically, in works that have employed a search method for estimating Δ, a given number of nonzero *δ*_*i *_in Δ is assumed (see for instance [20,25]). Therefore it was of interest to investigate the effect of this assumption on producing good estimates of Δ and *ε*_*gen*_. This can be tested for the simulated datasets in the demonstration, where the number of nonzero *δ*_*i *_is known. The *C*^1^*C*^2 ^was therefore applied to the simulated datasets both with an assumption about the number of nonzero *δ*_*i *_and without the assumption. For simplicity (however somewhat unrealistically), we assumed the correct number of nonzero *δ*_*i*_.

## Results of the testing

### Simulated datasets

The four simulated datasets in combination with the use of either the *C*^1^*C*^2 ^or repeated *K*-fold cross-validation for model choice and assessment, the GA or the brute-force search method, and either with or without the assumption of prior knowledge of the number of nonzero *δ*_*i *_constitute a two-level, five-factor, full factorial experimental design. The *C*^1^*C*^2 ^and the repeated *K*-fold cross-validation were applied four times to each factor combination, thus providing four replicates of the whole demonstration for the simulated data. The design can be analyzed within the normal linear model

(6) *w*_*iv *_= *γ*_0 _+ *γ*_1 _*z*_1*i *_+ *γ*_2_*z*_2*i *_+ *γ*_3_*z*_3*i *_+ *γ*_4_*z*_4*i *_+ *γ*_5_*z*_5*i *_+ η *i *(*i *= 1,...,128),

where *z*_*ji*_, *j *= 1,2,3,4,5, are factors corresponding to *C*^1^*C*^2 ^or repeated *K*-fold cross-validation model choice and assessment, brute force or GA search, Σ_20 _= ***I***_20 _or Σ_20 _= ***S***_20 _in the multivariate normal distribution from which the data was sampled, 200 or 15 observations, and assuming three nonzero *δ*_*i *_or no such assumption, respectively. *i *goes from 1 to 128 in (6) as there are 32 factor combinations in four replicates. *w*_*iv*_, *v *= 1,2,3, are response variables defined according to the following: the Euclidean norm *w*_*i*1 _= ${\left\|\bar{\hat{\Delta }}-\Delta \right\|}_{i}$ was used to measure how well Δ on average was estimated, *w*_*i*2 _= ${\bar{\hat{\lambda }}}_{i}$ was used as a response variable in the *λ *case (as the correct choice of *λ *is not known), and *w*_*i*3 _= ${\bar{\left|{\hat{\varepsilon }}_{gen}-{\tilde{\varepsilon }}_{gen}\right|}}_{i}$ was used to measure how well the generalization error *ε *on average was estimated; ${\hat{\varepsilon }}_{gen}$ denotes the estimate of *ε*_*gen *_for given values of *r *and *k*; ${\tilde{\varepsilon }}_{gen}=\frac{1}{N}\sum_{j=1}^{N}{\left(y_{j,ext}-{\hat{y}}_{j,ext}(x_{j,ext},M_{l})\right)}^{2}$ denotes the generalization error estimate obtained by using the corresponding choice of model, $M_{l}$, to predict the response values in a large (*N *= 500,000) external test set, generated in the same way as the dataset used for choosing the model and estimating ${\hat{\varepsilon }}_{gen}$; the bar denotes the average over the *R*·*K *individual estimates. The generalization error can be decomposed into three parts: one irreducible error (corresponding to the error added when simulating the data), the squared bias, and the variance. The latter two are dependent on the model choice and consequently the generalization error is dependent on the model choice. We here assume that the large-sample estimate of the generalization error, ${\tilde{\varepsilon }}_{gen}$, closely represents the true generalization error, *ε*, for a given model choice.

The results for choosing a model $M_{l}^{ridge}\in M^{ridge}$ for the simulated datasets are available in Additional file 5, where $\left\|\bar{\hat{\Delta }}-\Delta \right\|$, $\bar{\hat{\lambda }}$, and $\bar{\left|{\hat{\varepsilon }}_{gen}-{\tilde{\varepsilon }}_{gen}\right|}$ are tabulated for each factor combination and replicate. The parameter estimates for fitting the model (6) using $\left\|\bar{\hat{\Delta }}-\Delta \right\|$, $\bar{\hat{\lambda }}$, and $\bar{\left|{\hat{\varepsilon }}_{gen}-{\tilde{\varepsilon }}_{gen}\right|}$ as response variables are shown in Tables 1, 2, and 3, respectively. All fitted models were highly significant (*F*_5,122 _= 26.1, *p*-value < 2.2 × 10^-16 ^with $\left\|\bar{\hat{\Delta }}-\Delta \right\|$ as response; *F*_5,122 _= 47.7, *p*-value < 2.2 × 10^-16 ^with $\bar{\hat{\lambda }}$ as response; and *F*_5,122 _= 12.1, *p*-value = 1.6 × 10^-9 ^with $\bar{\left|{\hat{\varepsilon }}_{gen}-{\tilde{\varepsilon }}_{gen}\right|}$ as response); residual plots showed no large deviations from the assumptions of normality of error distribution (an asymptotic normal distribution of the response variables is warranted by the central limit theorem), homoscedasticity, and independent errors (data not shown). A few outliers were however observed, probably resulting from "unfortunate" data partitions.

**Table 1:** Coefficient estimates of model (6) with *w*_*i*1 _= ${\left\|\bar{\hat{\Delta }}-\Delta \right\|}_{i}$, *i *= 1, ..., 128, as a response variable.

	**Estimate**	**Std.Error**	**t-value**	**Pr(>|t|)**
intercept	-0.01992	0.04606	-0.433	0.6661
c1c2	-0.04337	0.03761	-1.153	0.2511
ga	0.15683	0.03761	4.170	5.72e-05
cor	0.07211	0.03761	1.918	0.0575
15	0.21324	0.03761	5.670	9.75e-08
all	0.32754	0.03761	8.710	1.78e-14

**c1c2 **– the *C*^1^*C*^2 ^was used (as opposed to repeated *K*-fold cross-validation), **ga **– the GA search method was used (as opposed to the brute force search method), **cor **– correlated independent variables in the dataset (as opposed to uncorrelated,) **15 **– *n *= 15 observations in the dataset (as opposed to *n *= 200), **all **– no assumption regarding the number of nonzero *δ*_*i *_(as opposed to the assumption of three *δ*_*i *_= 1).

**Table 2:** Coefficient estimates of model (6) with *w*_*i*2 _= ${\bar{\hat{\lambda }}}_{i}$, *i *= 1, ..., 128 as a response variable.

	**Estimate**	**Std.Error**	**t-value**	**Pr(>|t|)**
Intercept	0.02864	0.04732	0.605	0.546181
c1c2	-0.04804	0.03863	-1.244	0.216065
ga	-0.07329	0.03863	-1.897	0.060193
cor	0.56058	0.03863	14.510	< 2e-16
15	0.14307	0.03863	3.703	0.000321
all	0.11504	0.03863	2.977	0.003506

See Table 1 for notation explanation.

**Table 3:** Coefficient estimates of model (6) with *w*_*i*3 _= ${\bar{\left|{\hat{\varepsilon }}_{gen}-{\tilde{\varepsilon }}_{gen}\right|}}_{i}$, *i *= 1, ..., 128 as a response variable.

	**Estimate**	**Std.Error**	**t-value**	**Pr(>|t|)**
intercept	0.034642	0.004841	7.157	6.73e-11
c1c2	-0.024003	0.003952	-6.073	1.47e-08
ga	-0.001149	0.003952	-0.291	0.771710
cor	0.006198	0.003952	1.568	0.119423
15	0.013469	0.003952	3.408	0.000888
all	-0.012089	0.003952	-3.059	0.002732

See Table 1 for notation explanation.

### Selwood dataset

Applying the *C*^1^*C*^2 ^to the Selwood dataset yielded $\hat{\Delta }$ that on average contained 11.4 ± 3.5 nonzero ${\hat{\delta }}_{i}$. The 11 most frequently picked variables were the partial atomic charge for atoms ATCH1, ATCH3, and ATCH6; electrophilic superdelocalizability for atom ESDL3; the van der Waal's volume VDWVOL; the surface area SURF_A; the principal moments of inertia MOFI_Y and MOFI_Z; the principal ellipsoid axis PEAX_X; the partition coefficient LOGP; and the sum of F substituent constant SUM_F (see [19] for more details about the variables). The estimation of *λ *by $\bar{\hat{\lambda }}$ was 2.50 ± 0.09 and the estimation of the generalization error by ${\bar{\hat{\varepsilon }}}_{gen}$ was 0.42 ± 0.038, where ${\bar{\hat{\varepsilon }}}_{gen}$ is an average over the *R*·*K *${\hat{\varepsilon }}_{gen}$ produced in the *C*^1^*C*^2^.

Applying repeated *K*-fold cross-validation for model choice and assessment to the Selwood dataset gave on average 14.1 ± 4.8 selected variables. The 14 most frequently picked variables included the same 11 variables picked by the *C*^1^*C*^2 ^(see above) plus DIPMOM (the dipole moment), ATCH7 (partial charge of atom 7), and DIPV_Y (the dipole moment vector in the Y-direction). The estimation of *λ *by $\bar{\hat{\lambda }}$ was 3.01 ± 0.22 and the estimation of the generalization error by $\bar{\hat{\varepsilon }}$ was 0.35 ± 0.041, where $\bar{\hat{\varepsilon }}$ is an average over the *R*·*K *$\hat{\varepsilon }$ produced in the repeated *K*-fold cross-validation.

## Implementation

Computer programs to implement the *C*^1^*C*^2 ^were written in Java (Sun Microsystems [26]) as a part of the library P, that will serve as the data analysis plugin for Bioclipse [27]. P is available under the GSPL license from the website [28]; it is open source and free for academics. It has a modular architecture that enables plugging in new features, including modeling methods, model selection criteria, and search procedures. P relies on a modified version of the JGap library (available from the website [29]) for the genetic algorithm computations (the modifications are available under the LGPL license from the website [30]). The R-package, pvclust [31,32], was used for the cluster analysis (see Discussion).

## Discussion

### Simulated datasets

The model (6) fitted to *w *= $\left\|\bar{\hat{\Delta }}-\Delta \right\|$ (see Table 1) showed a relatively clear significant difference (on the 90% level) in average Δ estimates depending on whether the data came from a multivariate normal distribution with Σ_20 _= ***I***_20 _or Σ_20 _= ***S***_20_. Furthermore, we observed significant positive impacts on average Δ estimates with more observations and knowledge about the number of nonzero *δ*_*i*_. All these findings were expected; highly correlated variables should provide worse estimates of Δ, whereas more observations and trustworthy prior knowledge should provide better estimates. A significant improvement in average Δ estimates was observed when using the brute-force search compared to the GA. The GA on average selected slightly more variables than needed and than what the brute-force method did. No clear significant difference could be seen between using the *C*^1^*C*^2 ^rather than repeated *K*-fold cross-validation.

It can be shown that the optimal choice of *λ *(in terms of minimized expected generalization error) tends to zero as the number of observations tends to infinity and decreases with decreasing number of variables (see for instance [33]) and with decreasing correlations between the independent variables. The model (6) fitted with *w *= $\bar{\hat{\lambda }}$ as a response (see Table 2) showed that the average estimated *λ *was significantly smaller for the data that came from a multivariate normal distribution with Σ_20 _= ***I***_20 _compared to Σ_20 _= ***S***_20_, when more observations were used, and when prior knowledge about the number of nonzero *δ*_*i *_in Δ was assumed. Although the true value of *λ *is not known, these results are thus consistent with theory and provide evidence that both the *C*^1^*C*^2 ^and repeated *K*-fold cross-validation gave reasonable estimates of *λ *in the demonstration. However, the average *λ *estimates are not equal to zero for all orthogonal datasets, presumably due to the stochastic nature of the GA and to errors in ***y***_*n*_. No significant differences were observed between using the GA or the brute force search methods or between the *C*^1^*C*^2 ^and the repeated *K*-fold cross-validation.

**Figure 2:**
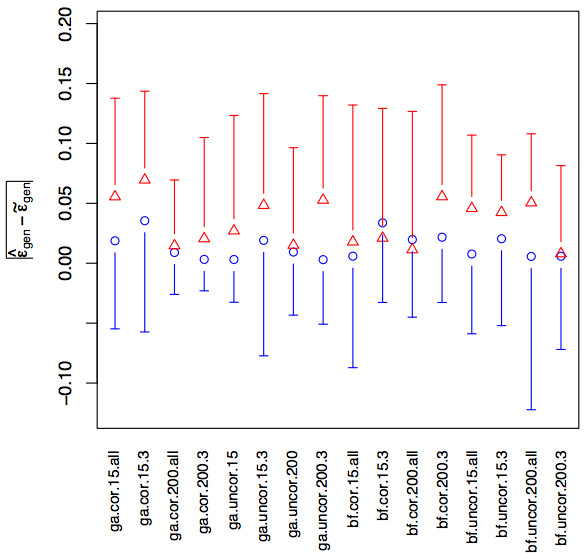
**Generalization errors obtained with the *C*^1^*C*^2 ^and repeated *K*-fold cross-validation**. The figure shows $\bar{\left|{\hat{\varepsilon }}_{gen}-{\tilde{\varepsilon }}_{gen}\right|}$, where ${\hat{\varepsilon }}_{gen}$ were produced using the *C*^1^*C*^2 ^(blue) and repeated *K*-fold cross-validation (red) for all other factor combinations in model (6). The plot is based on pooled $\bar{\left|{\hat{\varepsilon }}_{gen}-{\tilde{\varepsilon }}_{gen}\right|}$ over the four replicates for each method. The bars show the 95% confidence interval, calculated from the pooled results (the confidence intervals are only shown in one direction to avoid cluttering). The factor combinations in model (6) are coded as: **ga **– the GA search method was used, **bf **– the brute force search method was used, **uncor **– orthogonal independent variables in the dataset, **cor **– correlated independent variables in the dataset, **15 **– *n *= 15 observations in the dataset, **200 **– *n *= 200 observations in the dataset, **all **– no assumption regarding the number of nonzero *δ*_*i*_, **3 **– three *δ*_*i *_= 1 were assumed.

Fitting model (6) with *w *= $\bar{\left|{\hat{\varepsilon }}_{gen}-{\tilde{\varepsilon }}_{gen}\right|}$ as a response (see Table 3) showed that the average error estimates were significantly worsened with the assumption of a given number of nonzero *δ*_*i *_and that no significant difference was observed when using the GA or the brute force method, or when the independent variables in the dataset were correlated or not. These findings might seem confusing given that the assumption of a given number of nonzero *δ*_*i*_, the use of the brute-force search method, and uncorrelated independent variables all improved model selection. The findings can be explained by the fact that, in general, without an assumption of a given number of nonzero *δ*_*i*_, when using the GA for searching the model space, and when independent variables were correlated, more nonzero *δ*_*i *_are on average selected (see Table 1). Thus the chances of also selecting the correct ones improve. This implies that it is worse to estimate at least one *δ*_*i *_*= *1 with ${\hat{\delta }}_{i}$ = 0 than to estimate all *δ*_*i *_= 1 with ${\hat{\delta }}_{i}$ = 1 and at least one *δ*_*i *_= 0 with ${\hat{\delta }}_{i}$ = 1. This makes sense, because the former models are incorrect, whereas the latter ones contain the true model, but are inefficient due to their unnecessary large size. The average error estimates were significantly improved with a large number of observations and when the *C*^1^*C*^2 ^was employed to produce the estimates compared to when the repeated *K*-fold cross-validation was used (see Fig. 2 and Table 3). The latter result seems contradictory with that no clear difference was found between the average Δ estimates produced with the *C*^1^*C*^2 ^and those obtained with the repeated *K*-fold cross-validation (see above). It can however be explained by studying the *R*·*K *individual Δ estimates, where a clear (99% level) positive effect could be observed when using the *C*^1^*C*^2 ^compared to the repeated *K*-fold cross-validation. The *individual *Δ estimates were thus worse when repeated *K*-fold cross-validation was used, resulting in worse generalization error estimates. However *the average *Δ estimates from the respective method were almost the same. This observation is seconded by the higher confidence intervals of the average Δ estimates produced with repeated *K*-fold cross-validation (see Additional file 5). The finding that the *C*^1^*C*^2 ^produces more accurate generalization error estimates than repeated *K*-fold cross-validation is consistent with the results presented in for instance [9] and provides evidence for that a complete separation of the data used for model choice and the data used for model assessment is necessary to obtain better estimates of the generalization error.

### Selwood dataset

**Figure 3:**
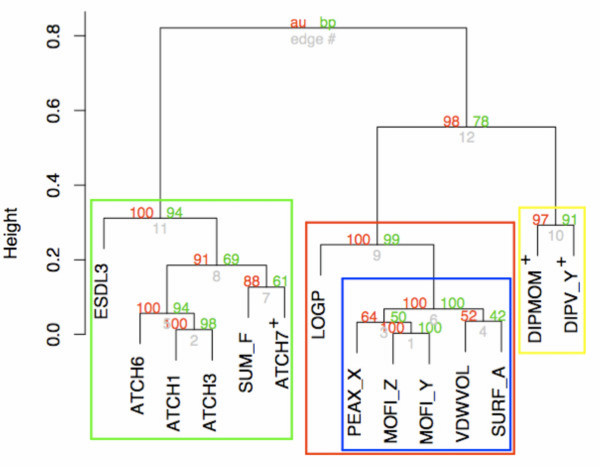
**Cluster dendrogram of the 14 selected variables from the Selwood dataset using repeated *K*-fold cross-validation**. Three distinct clusters can be noted (shown in red, green, and yellow rectangles). One sub-cluster can be seen within the red cluster (shown in a blue rectangle). The red and green numbers are *p*-values of a given cluster; they indicate how well the cluster is supported by data (see [31] for details). ^+^Additional variables selected by repeated *K*-fold cross-validation compared to the *C*^1^*C*^2^.

The result of estimating Δ was, expectedly, less clear when applying the *C*^1^*C*^2 ^and repeated *K*-fold cross-validation to real-world data; the Selwood dataset is particularly difficult to model due to the extremely high correlations between variables (many variable pairs have correlation coefficients > 0.95), the low observation-to-variable ratio, and deviations from the linearity and homoscedasticity assumptions (see [20]). 11.4 out of the 53 variables were on average selected by the *C*^1^*C*^2 ^and 14.1 by *K*-fold cross-validation. Interestingly, the 11 most frequently picked variables selected by the *C*^1 ^*C*^2 ^is a proper subset of the 14 most recurrently selected variables by *K*-fold cross-validation. Hierarchically clustering the 14 most frequently picked variables chosen by *K*-fold cross-validation (which thus includes the 11 variables selected most often by the *C*^1^*C*^2^) using the absolute correlation as a distance measure revealed three distinct clusters and one subcluster (see Fig. 3). Good models (in terms of estimated generalization error) for the Selwood dataset can be achieved by selecting LOGP and one variable from the set of variables in the blue subcluster (PEAX_X, MOFI_Y, MOFI_Z, VDWVOL, and SURF_A) and one from the set of variables in the green cluster (ESDL3, ATCH1, ATCH3, ATCH6, ATCH7, and SUM_F). LOGP appears to be sufficiently different from the rest of the variables in the red cluster to improve model performance. The variables in the respective blue and green clusters are highly correlated and it is sufficient to have one variable from each cluster in a model.

Models containing LOGP and one variable each from the green and blue clusters have high predictive power and comply to the QUICK rules for credible predictive models proposed previously [21]. Furthermore, these models have been found credible in the works of others [20,25,34]. The *C*^1^*C*^2 ^chose 11 variables belonging to the green, red, and blue clusters, whereas *K*-fold cross-validation chose an additional three variables: ATCH7 in the green cluster, and DIPMOM and DIPV_Y belonging to the third distinct (yellow) cluster. Variables from the yellow cluster do not improve the internal predictive ability when testing models containing LOGP and one variable from the respective green, blue, and yellow clusters on the whole Selwood dataset (data not shown); this result is supported by the work of Nicolotti and Carotti (see Table 1 in [20]). More variables in the Selwood dataset were thus on average selected with repeated *K*-fold cross-validation than when using the *C*^1^*C*^2 ^(the difference was significant on the 80% level, tested by a one-sided Welch's *t*-test), including two that not seem to improve the predictive ability of the models. The generalization estimates obtained with the *K*-fold cross-validation was lower than those obtained with the *C*^1^*C*^2 ^(significant on the 70% level). Although these differences are not highly significant, it is tantalizing to arrive at the conclusion that the models selected by repeated *K*-fold cross-validation in this particular case are more prone to overfitting and that this is reflected in the lower generalization error estimates.

### The *C*^1^*C*^2^

We have here introduced the *C*^1^*C*^2 ^framework for simultaneous model choice and assessment. The main idea is a complete separation of the choice of a model and its assessment in terms of the data used for each task. The *C*^1^*C*^2 ^was applied to the problem of choosing a model, $M_{l}^{ridge}\in M^{ridge}$. Previously, others have described methods that, within the linear model, tackle the problem of regression coefficient shrinkage and variable selection simultaneously, for example, the lasso [35]. However, the *C*^1^*C*^2 ^framework is general and is easily applied to other settings. For instance, different choices of *C*^2 ^are favorable in different situations; Akaike's information criterion (AIC) [36] is known to be consistent within the linear model if the true model is very complex, whereas BIC is favorable within linear models of finite dimension [37], and cross-validation is preferable to use in situations where the degrees of freedom of a model is difficult to define, and so forth. The search method can also be tailored to the problem at hand; for instance, brute-force methods are advantageous for small problems, whereas GAs are faster and thus applicable to larger problems. Moreover, if required, M can be chosen to contain nonlinear models, *L *can be chosen to be exponential in order to increase the penalty on outliers, and instead of using the search method to produce an estimate of Δ (as we did in the demonstration) we can let ***G ***contain a dedicated variable selection method. The cost of this generality is uncharacterized convergence rates (in finite time) of the parameter estimates, which is coupled to the need of employing a general search method (like a GA) rather than solving standard convex problems. Running the *C*^1^*C*^2 ^*R *times enables averaging of estimates and calculation of confidence intervals, but renders problems in choosing which out of the *R *models to use for interpretation and future predictions. A potential remedy to these problems is, instead of choosing *a *model, to employ *all *chosen models in a stacking-like schema (see [38] for details on stacking). Testing this idea and further testing of the *C*^1^*C*^2 ^for other choices of M, ***G***, *L*, *C*^1^, *C*^2^, and *S *will be pursued in future research.

## Conclusion

We have presented some evidence that suggests that the *C*^1^*C*^2 ^works well in terms of choosing the correct model and produce good estimates of the generalization error. It was demonstrated to perform well within a penalized linear model, even for "difficult" datasets with highly correlated independent variables, a low observation-to-variable ratio, and deviations from model assumptions (see Table 4 for a summary of the findings in the demonstrations). However, more research is needed to fully assess the methods performance for more general, for instance nonlinear, models and to provide theoretical insight to frameworks such as the *C*^1^*C*^2^. The *C*^1^*C*^2 ^is general and reasonable choices of M, ***G***, *L*, *C*^1^, *C*^2^, and *S *help in achieving as unbiased estimates with as low a variance to as low a computational cost as possible. A framework that completely separates model choice from assessment in terms of used data, like the *C*^1^*C*^2^, should always be employed for model selection and assessment in order to avoid positive bias in the generalization error estimates and, ultimately, to avoid false conclusions and using dubious models to direct further research.

**Table 4:** Summary of the demonstrations of the *C*^1^*C*^2^.

Both the *C*^1^*C*^2 ^and repeated *K*-fold cross-validation performed well at finding the true Δ (even when independent variables are highly correlated and when *n *<*p*).
The *C*^1^*C*^2 ^and repeated *K*-fold cross-validation produced reasonable estimates of *λ*.
Prior information about the number of important independent variables improves model choice but can reduce the accuracy of generalization error estimates.
Correlated independent variables and using the genetic algorithm worsened the model choice significantly, but not the generalization error estimates.
The *C*^1^*C*^2 ^compares favourably with repeated *K*-fold cross-validation for assessing the generalization error.

*n *denotes the number of observations in a dataset, *p *the number of variables, Δ represents a given subset of the *p *variables, and *λ *the ridge regression parameter.

## Methods

### Bayesian Information Criterion (BIC)

Suppose we have a set of candidate models, M and corresponding model parameters, *θ*_*m*_, and we wish to choose the best model among M. Assuming we have a prior distribution, P(*θ*_*m*_|$M_{m}$) for the parameters of each model, $M_{m}\in M$, the posterior probability of a given model is:

$$\begin{array}{l} \text{P}(M_{m}|D)\propto \text{P}(M_{m})\cdot \text{P}(D|M_{m})\propto \\ \text{P}(M_{m})\cdot \int \text{P}(D|\theta_{m},M_{m})\text{P}(\theta_{m}|M_{m})d\theta_{m}. \end{array}$$

To choose a model in a Bayesian setting, the posterior odds, given by:

(7) $$\frac{\text{P}(M_{l}|D)}{\sum_{m\text{=1}}^{M}\text{P}(M_{m}|D)}=\frac{\text{P}(M_{l})\text{P}(D|M_{l})}{\sum_{m\text{=1}}^{M}\text{P}(M_{m})\text{P}(D|M_{m})}$$

are formed for all models $M_{l}\in M$, and $M_{l}$ is picked to maximize equation (7). If all models in M are given equal prior probabilities, the problem of choosing the model $M_{l}$ is reduced to calculating the integrals, P(***D***|$M_{m}$). It can be shown [39,40] that the Bayesian Information Criterion (BIC) approximates the logarithm of this integral with an *O*(1) error term, that is:

$$\log\text{P}(D|M_{m})=BIC+O(1),$$

where

$$BIC=\log\text{P}(D|{\hat{\theta }}_{m},M_{m})-\frac{df_{m}}{2}\log n.$$

In the latter expression, ${\hat{\theta }}_{m}$ is the maximum likelihood estimate, *df*_*m *_is the number of free parameters in model $M_{m}$ (note that this in general is not equal to the number of parameters in the model), and *n *is the number of observations [17]. Thus, BIC is an approximation to the Bayes solution, but valid outside the Bayesian context. This is true because the leading terms in the approximation do not depend on the prior densities of the model parameters, *θ*_*m*_. BIC is, as opposed to nonparametric approaches such as cross-validation, model based and therefore relies on the assumptions made in the modeling. BIC is derived under the assumption that the data comes from a distribution in the exponential family (see [41] for more about the assumptions behind BIC and a comparison with Akaike's Information Criterion).

### Ridge regression

The ordinary least squares (OLS) estimator of the regression coefficients *β *in the standard linear model is efficient (i.e. has the minimum possible variance) within the class of linear and unbiased estimators. However, when the independent variables are correlated, the variance of the OLS estimator is generally high. In these situations, ridge regression [42] can yield improved parameter estimates by minimizing a penalized residual sum of squares, given by: *RSS *(*λ*) = (***y *- *X****β*)^*T *^(***y *- *X****β*) + *λβ*^T ^*β*. Finding the minimum of this expression gives the ridge solution: ${\hat{\beta }}^{ridge}$ = (***X***^*T *^***X *+ *λI***)^-1 ^***X***^*T *^***y***, where ***I ***is the *p *× *p *identity matrix. The solution thus adds a positive constant to the diagonal of ***X*^*T *^*X ***before inversion; this makes the problem nonsingular, even if ***X*^*T *^*X ***is not of full rank. While this introduces bias into the coefficient estimates, variance is often greatly reduced.

Note that ${\hat{\beta }}^{ridge}$ is a linear function in ***y***, thus it is straightforward to define the effective degrees of freedom of the ridge regression fit, (df(*λ*)) as:

df (*λ*) = tr **[*X*(*X*^*T *^X + ***λ****I*)**^-1^**X**^*T*^**] **[16]. The degrees of freedom of the fit are needed for carrying out model selection according to, for instance, BIC. Linearity in ***y ***also enables easy implementation (no quadratic programming required as, for instance, is necessary with the lasso).

### Genetic algorithm (GA)

A GA (see [43] for more details) is a stochastic search technique for finding exact or approximate solutions to optimization and search problems. A typical genetic algorithm is defined by a genetic representation of a given solution (normally termed a *chromosome *in the GA context). That is, a vector, $w_{t}^{i}$ specifies the numerical representation of the *i*th chromosome at generation *t*, and an objective function (or *fitness function*), *f*($w_{t}^{i}$)→/ℝ/evaluates the fitness of a chromosome. The GA is initiated by setting up a random population that contains a number of trial chromosomes. New solutions are generated by mutation or recombination of existing solutions and are selected for the next generation with a probability given by: $p(w_{t}^{i})=f(w_{t}^{i})/\sum_{j}^{a}f(w_{t}^{i})$. The process is continued through a number of generations until an optimal or acceptable solution has been found. Genetic algorithms of this type can be shown to converge with a probability of one to the global optimal solution as *t *→ ∞.

### Brute force search

A **brute force search **systematically tests an exhaustive list of all possible candidates for the solution to a given search or optimization problem and checks whether each candidate satisfies the problem's statement.

## Availability and requirements

• Project name: P

• Project homepage: 

• Operating systems: Platform independent (interpreted language)

• Programming language: Java

• Requirements: Java 5 or higher. A modified version of the JGAP package  for genetic algorithms. The modifications are distributed under the LGPL license and are available at . log4j, available from .

• Licence: GSPL (see )

• Restrictions to use for commercial purposes: licence needed

## Authors' contributions

ME devised and implemented the proposed *C*^1^*C*^2 ^framework. OS was involved in program design and aided with the implementation. JESW supervised the project. All authors read and approved the final manuscript.

## Supplementary Material

Additional file 1 The first of the four simulated datasets used in the demonstrations. This dataset with 15 observations was sampled from a multivariate normal distribution with mean ***0*_20 _**and covariance matrix ***I*_20 _**(see article for details). Click here for file

Additional file 2 The second of the four simulated datasets used in the demonstrations. This dataset with 200 observations was sampled from a multivariate normal distribution with mean ***0*_20 _**and covariance matrix ***I*_20 _**(see article for details). Click here for file

Additional file 3 The third of the four simulated datasets used in the demonstrations. This dataset with 15 observations was sampled from a multivariate normal distribution with mean ***0*_20 _**and covariance matrix ***I*_20 _**(see article for details). Click here for file

Additional file 4 The fourth of the four simulated datasets used in the demonstrations. This dataset with 200 observations was sampled from a multivariate normal distribution with mean ***0*_20 _**and covariance matrix ***S*_20 _**(see article for details). Click here for file

Additional file 5 ***C*^1^*C*^2 ^and repeated *K*-fold cross-validation estimates of Δ, *λ*, and *ε *for the simulated data**. The factor combinations in model (6) are coded as: **c1c2 **– the *C*^1^*C*^2 ^was used, **k-fold **– repeated *K*-fold cross-validation was used, **ga **– the GA search method was used, **bf **– the brute force search method was used, **15 **– *n *= 15 observations in the dataset, **200 **– *n *= 200 observations in the dataset, **uncor **– orthogonal independent variables in the dataset, **cor **– correlated independent variables in the dataset, **all **– no assumption regarding the number of nonzero *δ*_*i*_, **3 **– three *δ*_*i *_= 1 were assumed. All values are means ± 95% confidence intervals, assuming normal distributions for $\left\|\bar{\hat{\Delta }}-\Delta \right\|$, $\bar{\hat{\lambda }}$, and $\bar{\left|{\hat{\varepsilon }}_{gen,i}-{\tilde{\varepsilon }}_{gen,i}\right|}$. Click here for file
